# Involvement of Machine Learning for Breast Cancer Image Classification: A Survey

**DOI:** 10.1155/2017/3781951

**Published:** 2017-12-31

**Authors:** Abdullah-Al Nahid, Yinan Kong

**Affiliations:** School of Engineering, Macquarie University, Sydney, NSW 2109, Australia

## Abstract

Breast cancer is one of the largest causes of women's death in the world today. Advance engineering of natural image classification techniques and Artificial Intelligence methods has largely been used for the breast-image classification task. The involvement of digital image classification allows the doctor and the physicians a second opinion, and it saves the doctors' and physicians' time. Despite the various publications on breast image classification, very few review papers are available which provide a detailed description of breast cancer image classification techniques, feature extraction and selection procedures, classification measuring parameterizations, and image classification findings. We have put a special emphasis on the Convolutional Neural Network (CNN) method for breast image classification. Along with the CNN method we have also described the involvement of the conventional Neural Network (NN), Logic Based classifiers such as the Random Forest (RF) algorithm, Support Vector Machines (SVM), Bayesian methods, and a few of the semisupervised and unsupervised methods which have been used for breast image classification.

## 1. Introduction

The cell of the body maintains a cycle of regeneration processes. The balanced growth and death rate of the cells normally maintain the natural working mechanism of the body, but this is not always the case. Sometimes an abnormal situation occurs, where a few cells may start growing aberrantly. This abnormal growth of cells creates cancer, which can start from any part of the body and be distributed to any other part. Different types of cancer can be formed in human body; among them breast cancer creates a serious health concern. Due to the anatomy of the human body, women are more vulnerable to breast cancer than men. Among the different reasons for breast cancer, age, family history, breast density, obesity, and alcohol intake are reasons for breast cancer.

**Figure 1:**
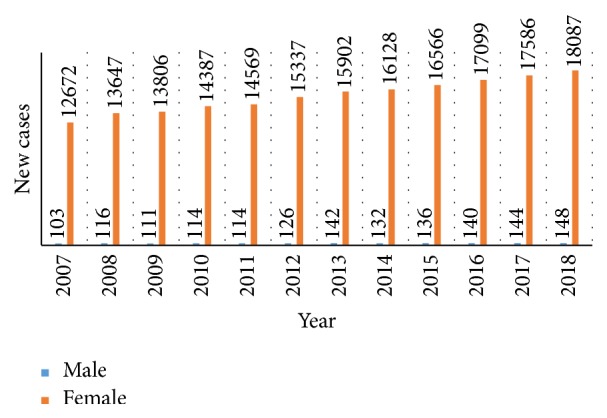
Number of new people facing cancer in Australia from 2007 to 2018 [5].

Statistics reveal that in the recent past the situation has become worse. As a case study, Figure 1 shows the breast cancer situation in Australia for the last 12 years. This figure also shows the number of new males and females to start suffering from breast cancer. In 2007, the number of new cases for breast cancer was 12775, while the expected number of new cancer patients in 2018 will be 18235. Statistics show that, in the last decade, the number of new cancer disease patients increased every year at an alarming rate.

**Figure 2:**
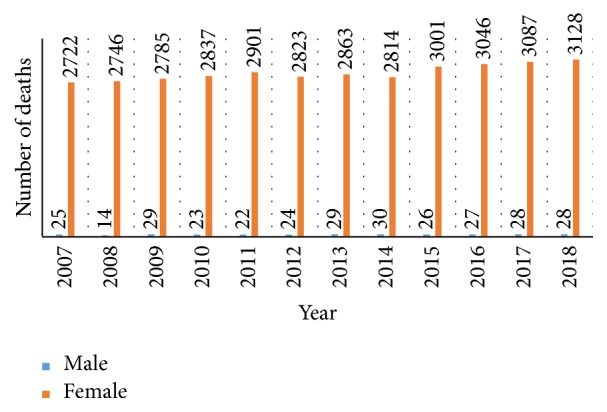
Number of people dying due to cancer in Australia from 2007 to 2018 [5].

Figure 2 shows the number of males and females facing death due to breast cancer. It is predicted that in 2018 around 3156 people will face death; among them 3128 will be women which is almost 99.11% of the overall deaths due to breast cancer.

**Figure 3:**
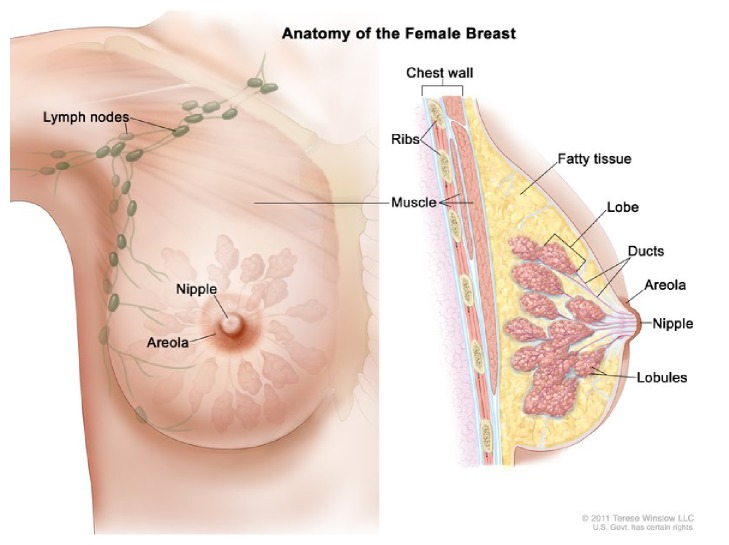
Anatomy of the female breast images (for the National Cancer Institute 2011; Terese Winslow, US Government, has certain rights).

Women's breasts are constructed by lobules, ducts, nipples, and fatty tissues. Milk is created in lobules and carried towards nipple by ducts. Normally epithelial tumors grow inside lobules as well as ducts and later form cancer inside the breast [1]. Once the cancer has started it also spreads to other parts of the body.  Figure 3 shows the internal construction from a breast image.

Breast cancer tumors can be categorized into two broad scenarios.

*(i) Benign (Noncancerous)*. Benign cases are considered as noncancerous, that is, non-life-threatening. But on a few occasions it could turn into a cancer status. An immune system known as “sac” normally segregates benign tumors from other cells and can be easily removed from the body.

*(ii) Malignant (Cancerous)*. Malignant cancer starts from an abnormal cell growth and might rapidly spread or invade nearby tissue. Normally the nuclei of the malignant tissue are much bigger than in normal tissue, which can be life-threatening in future stages.

Cancer is always a life-threatening disease. Proper treatment of cancer saves people's lives. Identification of the normal, benign, and malignant tissues is a very important step for further treatment of cancer. For the identification of benign and malignant conditions, imaging of the targeted area of the body helps the doctor and the physician in further diagnosis. With the advanced modern photography techniques, the image of the targeted part of the body can be captured more reliably. Based on the penetration of the skin and damage of the tissue medical photography techniques can be classified into two groups.

*(i) Noninvasive*. (a) Ultrasound: this photography technique uses similar techniques to SOund Navigation And Ranging (SONAR) which operates in the very-high-frequency domain and records the echos of that frequency, invented by Karl Theodore Dussik [2]. An ultrasound image machine contains a Central Processing Unit (CPU), transducer, a display unit, and a few other peripheral devices. This device is capable of capturing both 2D and 3D images. Ultrasound techniques do not have any side-effects, with some exceptions like production of heat bubbles around the targeted tissue. (b) X-ray: X-rays utilize electromagnetic radiation, invented by Wilhelm Conrad Roentgen in 1895. The mammogram is a special kind of X-ray (low-dose) imaging technique which is used to capture a detailed image of the breast [3]. X-rays sometimes increase the hydrogen peroxide level of the blood, which may cause cell damage. Sometimes X-rays may change the base of DNA. (c) Computer Aided Tomography (CAT): CAT, or in short CT imaging, is advanced engineering of X-ray imaging techniques, where the X-ray images are taken at different angles. The CT imaging technique was invented in 1970 and has been mostly used for three-dimensional imaging. (d) Magnetic Resonance Imaging (MRI): MRI is a noninvasive imaging technique which produces a 3D image of the body, invented by Professor Sir Peter Marsfield, and this method utilizes both a magnetic field as well as radio waves to capture the images [4]. MRI techniques take longer to capture images, which may create discomfort for the user. Extra cautions need to be addressed to patients who may have implanted extra metal.

**Figure 4:**
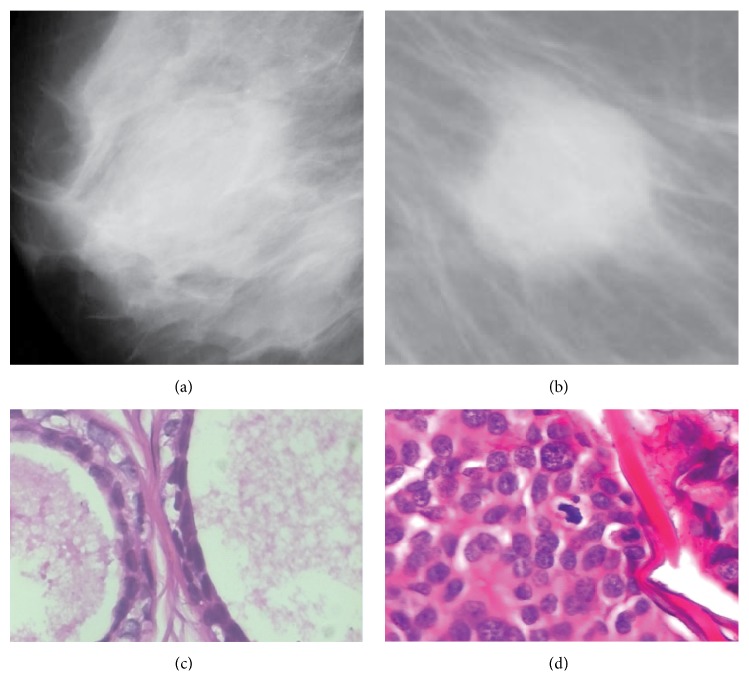
(a, b) show mammogram benign and malignant images (examples of noninvasive image) and (c, d) show histopathological benign and malignant images (examples of invasive image).

*(ii) Invasive*. (a) Histopathological images (biopsy imaging): histopathology is the microscopic investigation of a tissue. For histopathological investigation, a patient needs to go through a number of surgical steps. The photographs taken from the histopathological tissue provide histopathological images (see Figure 4).

## 2. Breast Image Classification

**Figure 5:**
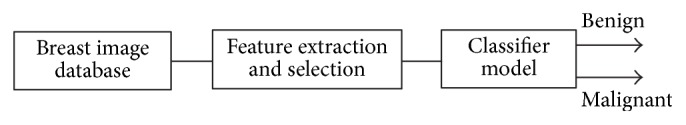
A very basic breast image classification model.

Various algorithms and investigation methods have been used by researchers to investigate breast images from different perspectives depending on the demand of the disease, the status of the disease, and the quality of the images. Among the different tasks, for breast image classification, machine learning (ML) and the Artificial Intelligence (AI) are heavily utilized. A general breast image classifier consists of four stages (see Figure 5):

- Selection of a breast database
- Feature extraction and selection
- Classifier model
- Performance measuring parameter
- Classifier output.

Figure 5 shows a very basic breast image classifier model.

### 2.1. Available Breast Image Databases

Doctors and physicians are heavily reliant on the ultrasound, MRI, X-ray, and so forth images to find the breast cancer present status. However, to ease the doctors' work, some research groups are investigating how to use computers more reliably for breast cancer diagnostics. To make a reliable decision about the cancer outcome, researchers always base their investigation on some well-established image database. Various organizations have introduced sets of images databases which are available to researchers for further investigation.  Table 1 gives a few of the available image databases, with some specifications.

**Figure 6:**
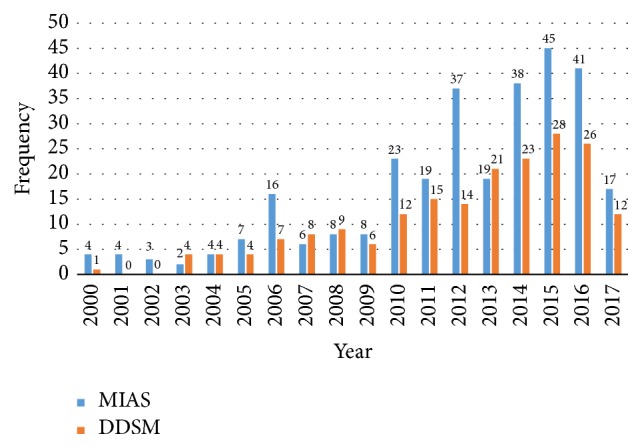
Number of papers published based on MIAS and DDSM databases.

The image formats of the different databases are different. Few of the images contained images in JPEG format and few databases contained DICOM-format data. Here the MIAS, DDSM, and Inbreast databases contain mammogram images. According to the Springer (http://www.springer.com), Elsevier (https://www.elsevier.com), and IEEE (http://www.ieeexplore.ieee.org) web sites, researchers have mostly utilized the MIAS and DDSM databases for the breast image classification research. The number of conference papers published for the DDSM and MIAS databases is 110 and 168, respectively, with 82 journal papers published on DDSM databases and 136 journal papers published using the MIAS database. We have verified these statistics on both Scopus (https://www.scopus.com) and the Web of Science database (http://www.webofknowledge.com).  Figure 6 shows the number of published breast image classification papers based on the MIAS and DDSM database from the years 2000 to 2017.

Histopathological images provide valuable information and are being intensively investigated by doctors for finding the current situation of the patient. The TCGA-BRCA and BreakHis databases contain histopathological images. Research has been performed in a few experiments on this database too. Among these two databases, BreakHis is the most recent histopathological image database, containing a total of 7909 images which have been collected from 82 patients [6]. So far around twenty research papers have been published based on this database.

### 2.2. Feature Extraction and Selection

**Figure 7:**
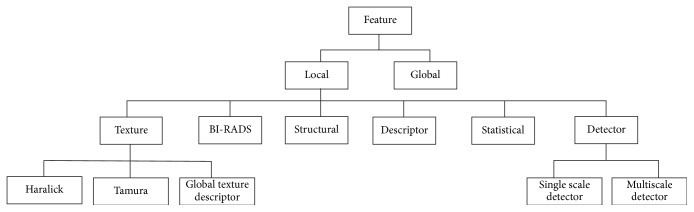
Classification of features for breast image classification.

An important step of the image classification is extracting the features from the images. In the conventional image classification task, features are crafted locally using some specific rules and criteria. However, the-state-of-the-art Convolutional Neural Network (CNN) techniques generally extract the features globally using kernels and these Global Features have been used for image classification. Among the local features, texture, detector, and statistical are being accepted as important features for breast image classification. Texture features actually represent the low-level feature information of an image, which provides more detailed information of an image that might be possible from histogram information alone. More specifically, texture features provide the structural and dimensional information of the color as well as the intensity of the image. Breast Imaging-Reporting and Data System (BI-RADS) is a mammography image assessment technique, containing 6 categories normally assigned by the radiologist. Feature detector actually provides information whether the particular feature is available in the image or not. Structural features provide information about the features structure and orientation such as the area, Convex Hull, and centroid. This kind of information gives more detailed information about the features. In a cancer image, it can provide the area of the nucleus or the centroid of the mass. Mean, Median, and Standard Deviation always provide some important information on the dataset and their distribution. This kind of features has been categorized as statistical features. The total hierarchy of the image feature extraction is resented in Figure 7. Tables 2 and 3 further summarize the local features in detail.

Features which are extracted for classification do not always carry the same importance. Some features may even contribute to degrading the classifier performance. Prioritization of the feature set can reduce the classifier model complexity and so it can reduce the computational time. Feature set selection and prioritization can be classified into three broad categories:

- Filter: the filter method selects features without evaluating any classifier algorithm.
- Wrapper: the wrapper method selects the feature set based on the evaluation performance of a particular classifier.
- Embedded: the embedded method takes advantage of the filter and wrapper methods for classifier construction.

**Figure 8:**
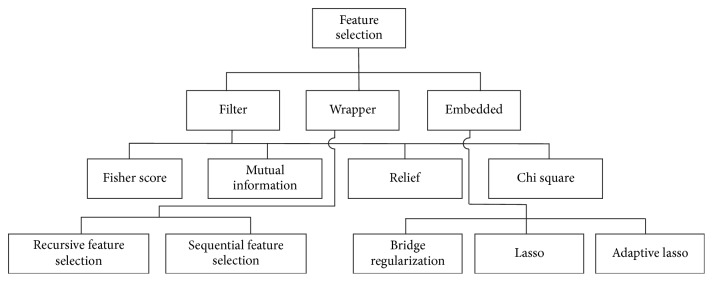
A summary of feature selection method.

Figure 8 shows a generalized feature selection method where we have further classified the filter method into Fisher Score, Mutual Information, Relief, and chi square methods. The embedded method has been classified into Bridge Regularization, Lasso, and Adaptive Lasso methods, while the wrapper method has been classified to recursive feature selection and sequential feature selection method.

### 2.3. Classifier Model

Based on the learning point of view, breast image classification techniques can be categorized into the following three classes [41]:

- Supervised
- Unsupervised
- Semisupervised.

These three classes can be split into Deep Neural Network (DNN) and conventional classifier (without DNN) and to some further classes as in Table 4.

### 2.4. Performance Measuring Parameter

**Figure 9:**
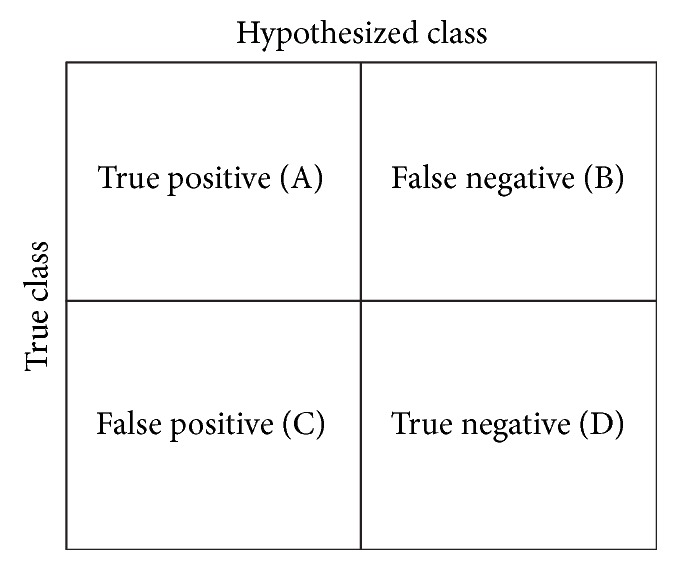
Confusion Matrix.

A Confusion Matrix is a two-dimensional table which is used to a give a visual perception of classification experiments [54]. The (*i*, *j*)th position of the confusion table indicates the number of times that the *i*th object is classified as the *j*th object. The diagonal of this matrix indicates the number of times the objects are correctly classified.  Figure 9 shows a graphical representation of a Confusion Matrix for the binary classification case.

Among the different classification performance properties, this matrix will provide following parameters:

(i) Recall is defined as Recall = TP/(TP + FN).
(ii) Precision is defined as Precision = TP/(TP + FP).
(iii) Specificity is defined as Specificity = TN/(TN + FP).
(iv) Accuracy is defined as ACC = (TP + TN)/(TP + TN + FP + FN).
(v) *F*-1 score is defined as *F*_1_ = (2 × Recall)/(2 × Recall + FP + FN).
(vi) Matthew Correlation Coefficient (MCC): MCC is a performance parameter of a binary classifier, in the range {−1 to +1}. If the MCC values trend more towards +1, the classifier gives a more accurate classifier and the opposite condition will occur if the value of the MCC trend towards the −1. MCC can be defined as
  (1) MCC=TP×TN−FP×FNTP+FPTP+FNTN+FPTN+FP.

## 3. Performance of Different Classifier Model on Breast Images Dataset

Based on Supervised, Semisupervised, and Unsupervised methods different research groups have been performed classification operation on different image database. In this section we have summarized few of the works of breast image classification.

### 3.1. Performance Based on Supervised Learning

In supervised learning, a general hypothesis is established based on externally supplied instances to produce future prediction. For the supervised classification task, features are extracted or automatically crafted from the available dataset and each sample is mapped to a dedicated class. With the help of the features and their levels a hypothesis is created. Based on the hypothesis unknown data are classified [55].

**Figure 10:**
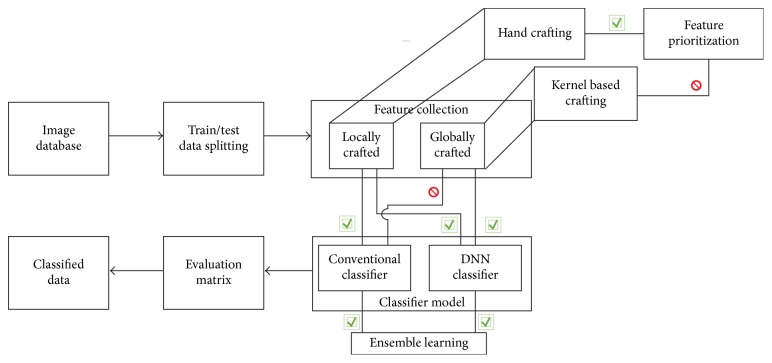
A generalized supervised classifier model.

Figure 10 represents an overall supervised classifier architecture. In general, the whole dataset is split into training and testing parts. To validate the data, some time data are also split into a validation part as well. After the data splitting the most important part is to find out the appropriate features to classify the data with the utmost Accuracy. Finding the features can be classified into two categories, locally and globally crafted. Locally crafted means that this method requires a hand-held exercise to find out the features, whereas globally crafted means that a kernel method has been introduced for the feature extraction. Handcrafted features can be prioritized, whereas Global Feature selection does not have this luxury.

#### 3.1.1. Conventional Neural Network

The Neural Network (NN) concept comes from the working principle of the human brain. A biological neuron consists of the following four parts:

- Dendrites
- Nuclease
- Cell body
- Axon.

**Figure 11:**
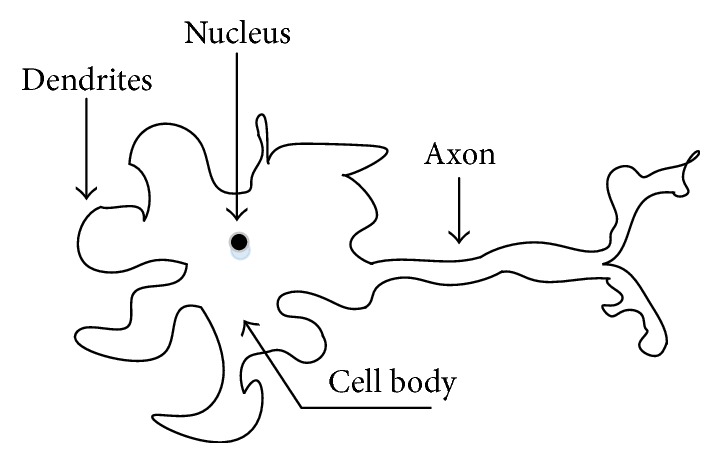
A model of a biological neuron.

**Figure 12:**
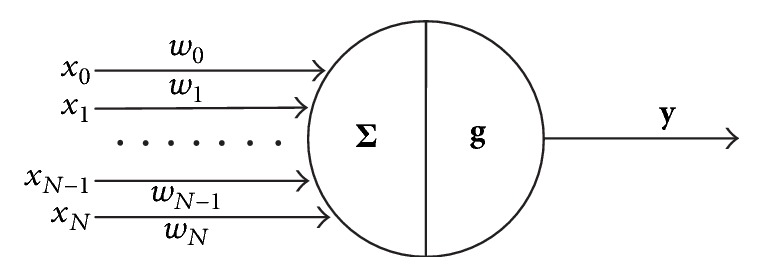
Working principle of a simple Neural Network technique.

Dendrites collect signals and axons carry the signal to the next dendrite after processing by the cell body as shown in Figure 11. Using the neuron working principle, the perceptron model was proposed by Rosenblatt in 1957 [56]. A single-layer perceptron linearly combines the input signal and gives a decision based on a threshold function. Based on the working principle and with some advanced mechanism and engineering, NN methods have established a strong footprint in many problem-solving issues.  Figure 12 shows the basic working principle of NN techniques.

In the NN model the input data **X** = {*x*_0_, *x*_1_,…, *x*_*N*_} is first multiplied by the weight data **W** = {*w*_0_, *w*_1_,…, *w*_*N*_} and then the output is calculated using

(2) $$\begin{array}{c} Y=g\left(\;\sum \;\;\right)\;where\sum \;=W\cdot X. \end{array}$$

Function **g** is known as the activation function. This function can be any threshold value or Sigmoid or hyperbolic and so forth. In the early stages, feed-forward Neural Network techniques were introduced [57]; lately the backpropagation method has been invented to utilize the error information to improve the system performance [58, 59].

The history of breast image classification by NN is a long one. To the best of my knowledge a lot of the pioneer work was performed by Dawson et al. in 1991 [60]. Since then, NN has been utilized as one of the strong tools for breast image classification. We have summarized some of the work related to NN and breast image classification in Tables 5, 6, and 7.

#### 3.1.2. Deep Neural Network

Deep Neural Network (DNN) is a state-of-the-art concept where conventional NN techniques have been utilized with advanced engineering. It is found that conventional NNs have difficulties in solving complex problems, whereas DNNs solve them with utmost Precision. However DNNs suffer from more time and computational complexity than the conventional NN.

- Convolutional Neural Network (CNN)
- Deep Belief Network (DBN)
- Generative Adverbial Network (GAN)
- Recurrent Neural Network (RNN)

*Convolutional Neural Network*. A CNN model is the combination of a few intermediate mathematical structures. This intermediate mathematical structure creates or helps to create different layers:

*(i) Convolutional Layer*. Among all the other layers, the convolutional layer is considered as the most important part for a CNN model and can be considered as the backbone of the model. A kernel of size *m* × *n* is scanned through the input data for the convolutional operation which ensures the local connectivity and weight sharing property.

*(ii) Stride and Padding*. In the convolutional operation, a filter scans through the input matrices. In each step how much position a kernel filter moves through the matrix is known as the stride. By default stride keeps to 1. With inappropriate selection of the stride the model can lose the border information. To overcome this issue the model utilizes extra rows and columns at the end of the matrices, and these added rows and columns contain all 0s. This adding of extra rows and columns which contain only zero value is known as zero padding.

*(iii) Nonlinear Operation*. The output of each of the kernel operations is passed through a rectifier function such as Rectified Linear Unit (ReLU), Leaky-ReLU, TanH, and Sigmoid. The Sigmoid function can be defined as

(3) $$\begin{array}{c} \sigma \left(\;x\;\right)=\frac{1}{\left(1+{exp}^{-x}\right)} \end{array}$$

and the tanh function can be defined as

(4) $$\begin{array}{c} \tanh\left(\;x\;\right)=\frac{\left({exp}^{x}-{exp}^{-x}\right)}{\left({exp}^{x}+{exp}^{-x}\right)}. \end{array}$$

**Figure 13:**
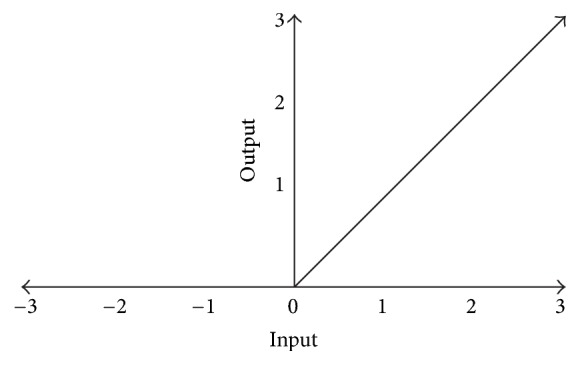
ReLU Operation.

However the most effective rectifier is ReLU. The ReLU method converts all the information into zero if it is less than or equal to zero and passes all the other data as is shown in Figure 13

(5) $$\begin{array}{c} \sigma \left(\;x\;\right)=\max\left(\;0,x\;\right). \end{array}$$

Another important nonlinear function is Leaky-RelU

(6) $$\begin{array}{c} Leaky\text{-}ReLU\left(\;x\;\right)=\sigma \left(\;x\;\right)+\alpha \min\left(\;0,x\;\right), \end{array}$$

where *α* is predetermined parameter which can be varied to give a better model.

*(iv) Subsampling*. Subsampling is the procedure of reducing the dimensionality of each of the feature maps of a particular layer; this operation is also known as a pooling operation. Actually it reduces the amount of feature information from the overall data. By doing so, it reduces the overall computational complexity of the model. To do this *s* × *s* patch units are utilized. The two most popular pooling methods are

(a) Max-Pooling
(b) Average Pooling.

**Figure 14:**
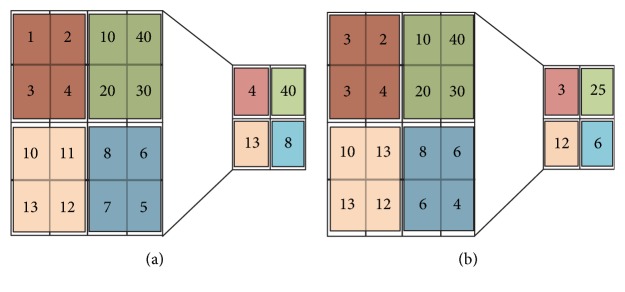
Max-Pooling and Average Pooling.

In Max-Pooling, only the maximum values within a particular kernel size are selected for further calculation. Consider an example of a 16 × 16 image as shown in Figure 14. A 2 by 2 kernel is applied to the whole image, 4 blocks in total, and produces a 4 × 4 output image. For each block of four values, we have selected the maximum. For instance, from blocks one, two, three, and four, maximum values 4, 40, 13, and 8 are selected, respectively, as they are the maximum in that block. For the Average Pooling operation, each kernel gives the output as average.

*(v) Dropout*. Regularization of the weight can reduce the outfitting problem. Randomly removing some neurons can regularize the overfilling problem. The technique of randomly removing neurons from the network is known as dropout.

*(vi) Soft-Max Layer*. This layer contains normalized exponential functions to calculate the loss function for the data classification.

**Figure 15:**
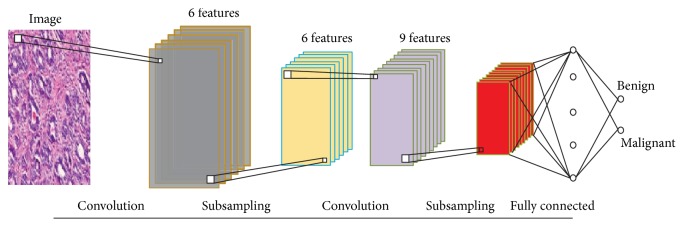
Work-flow of a Convolutional Neural Network.

Figure 15 shows a generalized CNN model for the image classification. All the neurons of the most immediate layer of a fully connected layer are completely connected with the fully connected layer, like a conventional Neural Network. Let *f*_*j*_^*l*−1^ represent the *j*th feature map at the layer *l* − 1. The *j*th feature map at the layer *l* can be represented as

(7) $$\begin{array}{c} f_{j}^{l}=\sigma \left(\;\overset{N^{l-l}}{\underset{i=1}{\sum }}f_{i}^{l-1}*k_{i,j}+b_{j}^{l}\;\right), \end{array}$$

where *N*^*l*−*l*^ represents the number of feature maps at the *l* − 1th layer, *k*_*i*,*j*_ represents the kernel function, and *b*_*j*_^*l*^ represents the bias at *l*, where *σ* performs a nonlinear function operation. The layer before the Soft-Max Layer can be represented as

(8) $$\begin{array}{c} h_{p}^{end}=w^{end}*h_{p}^{end-1}+b^{end}. \end{array}$$

As we are working on a binary classification, the Soft-Max regression normalized output can be represented as

(9) $$\begin{array}{c} {\bar{y}}_{p}=\frac{\exp\left(h_{p}^{end}\right)}{\sum_{p=1}^{2}\exp\left(h_{p}^{end}\right)}. \end{array}$$

Let *p* = 1 represent Benign class and *p* = 2 represent the Malignant class. The cross-entropy loss of the above function can be calculated as

(10) $$\begin{array}{c} L_{p}=-\ln\left(\;{\bar{y}}_{p}\;\right). \end{array}$$

Whichever group experiences a large loss value, the model will consider the other group as predicted class.

A difficult part of working on DNN is that it requires a specialized software package for the data analysis. Few research groups have been working on how effectively data can be analyzed by DNN from different perspectives and the demand.  Table 8 summarizes some of the software which is available for DNN analysis.

The history of the CNN and its use for biomedical image analysis is a long one. Fukushima first introduced a CNN named “necognitron” which has the ability to recognize stimulus patterns with a few shifting variances [113]. To the best of our knowledge, Wu et al. first classified a set of mammogram images into malignant and benign classes using a CNN model [78]. In their proposed model they only utilized one hidden layer. After that, in 1996 Sahiner et al. utilized CNN model to classify mass and normal breast tissue and achieved ROC scores of 0.87 [79]. In 2002, Lo et al. utilized a Multiple Circular Path CNN (MCPCNN) for tumor identification from mammogram images and obtained ROC scores of around 0.89. After an absence of investigation of the CNN model, this model regained its momentum after the work of Krizhevsky et al. [114]. Their proposed model is known as AlexNet. After this work a revolutionary change has been achieved in the image classification and analysis field. As an advanced engineering of the AlexNet, the paper titled “Going Deeper with Convolutions” by Szegedy [115] introduced the GoogleNet model. This model contains a much deeper network than AlexNet. Sequentially ResNet [116], Inception [117], Inception-v4, Inception-ResNet [118], and a few other models have recently been introduced.

Later, directly or with some advanced modification, these DNN models have been adapted for biomedical image analysis. In 2015, Fonseca et al. [81] classified breast density using CNN techniques. CNN requires a sufficient amount of data to train the system. It is always very difficult to find a sufficient amount of medical data for training a CNN model. A pretrained CNN model with some fine tuning can be used rather than create a model from scratch [119]. The authors of [119] did not perform their experiments on a breast cancer image dataset; however they have performed their experiments on three different medical datasets with layer-wise training and claimed that “retrained CNN along with adequate training can provide better or at least the same amount of performance.”

The Deep Belief Network (DBN) is another branch of the Deep Neural Network, which mainly consists of Restricted Boltzmann Machine (RBM) techniques. The DBN method was first utilized for supervised image classification by Liu et al. [120]. After that, Abdel-Zaher and Eldeib utilized the DBN method for breast image classification [121]. This field is still not fully explored for breast image classification yet. Zhang et al. utilized both RBM and Point-Wise Gated RBM (PRBM) for shear-wave electrography image classification where the dataset contains 227 images [97]. Their achieved classification Accuracy, Sensitivity, and Specificity are 93.40%, 88.60%, and 97.10%, respectively. Tables 9, 10, and 11 have summarized the most recent work for breast image classification along with some pioneer work on CNN.

#### 3.1.3. Logic Based Algorithm

**Figure 16:**
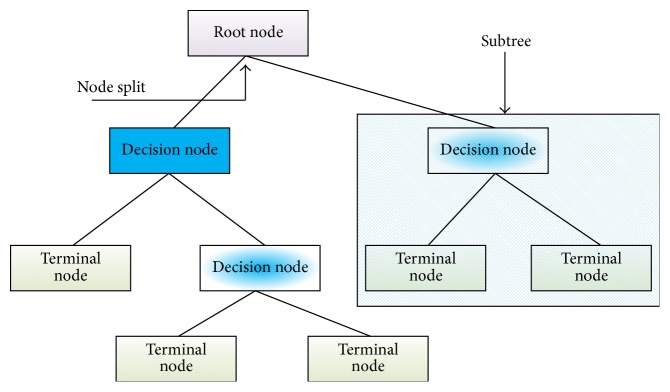
A general structure of a tree.

A Logic Based algorithm is a very popular and effective classification method which follows the tree structure principle and logical argument as shown in Figure 16. This algorithm classifies instances based on the feature's values. Along with other criteria, a decision-tree based algorithm contains the following features:

- Root node: a root node contains no incoming node, and it may or may not contain any outgoing edge
- Splitting: splitting is the process of subdividing a set of cases into a particular group. Normally the following criteria are maintained for the splitting:
  - information gain,
  - Gini index,
  - chi squared
- Decision node
- Leaf/terminal node: this kind of node has exactly one incoming edge and no outgoing edge. The tree always terminates here with a decision
- Pruning: pruning is a process of removing subtrees from the tree. Pruning performs to reduce the overfitting problem. Two kinds of pruning techniques are available:
  - prepruning,
  - postpruning.

Among all the tree based algorithms, Iterative Dichotomiser 3 (ID3) can be considered as a pioneer, proposed by Quinlan [149]. The problem of the ID3 algorithm is to find the optimal solution which is very much prone towards overfitting. To overcome the limitation of the ID3 algorithm the C4.5 algorithm has been introduced by Quinlan [150], where a pruning method has been introduced to control the overfitting problem. Pritom et al. [151] classified the Wisconsin breast dataset where they utilized 35 features. They have obtained 76.30% Accuracy, 75.10% False Positive Rate, and ROC score 0.745 when they ranked the features. Without ranking the features they obtained 73.70% Accuracy, 50.70% False Positive Rate, and ROC score value 52.80. Asri et al. [152] utilized the C4.5 algorithm for the Wisconsin database classification where they utilized 11 features and obtained 91.13% Accuracy.

Logic Based algorithms allow us to produce more than one tree and combine the decisions of those trees for an advanced result; this mechanism is known as an ensemble method. An ensemble method combines more than one classifier hypothesis together and produces more reliable results through a voting concept. Boosting and bagging are two well-known ensemble methods. Both boosting and bagging aggregate the trees. The difference is in bagging successive trees do not depend on the predecessor trees, where in the boosting method successive trees depend on the information gathered from the predecessor trees. Gradient boosting is a very popular method for data classification [153, 154]; however a state-of-the-art boosting algorithm such as “Extreme Gradient Boosting” (XGBoosting) is a very effective method for data classification [155]. Interestingly, there has not been a single paper published for breast image classification using the XGBoost algorithm. Along with the boosting method, different bagging methods are available; among them Random Forest (RF) is very popular where a large number of uncorrelated trees are aggregated together for a better prediction. Tables 12 and 13 summarize a set of papers where a Logic Based algorithm has been used for image classification.

#### 3.1.4. Support Vector Machine (SVM)

SVM were proposed by VC (Vepnick-Cherovorenkis). This technique does not require any prior distribution knowledge for the data classification task like Bayesian classification technique. In many practical situations, the distribution of the features is not available. In such cases, SVM can be used to classify the available data into the different classes.

**Figure 17:**
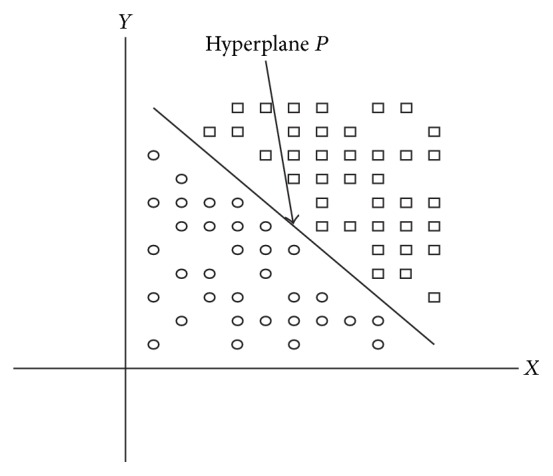
SVM finds the hyperplane which separates two classes.

Consider the set of two-dimensional data plotted in Figure 17. The symbol “∘” represents those data which belong to Class-1 and “□” represents data which belong to Class-2. A hyperplane (*P*) has been drawn which classifies the data into two classes. Interestingly, there will be “*n*” hyperplanes available which can separate the data.

Let **X** = {**X**_*i*_}, where {**X**_*i*_ ∈ *ℛ*^*n*^} (*i* = {1,2, 3,…, *l*}) is to be classified into two classes *ω* ∈ {*ω*^1^, *ω*^2^}. Suppose that the classes {*ω*^1^} and {*ω*^2^} are recognized as “+1” and “−1”. Classification of this data can be written

(11) $$\begin{array}{c} C=\left\{\;\left(\;X_{1},\omega_{1}\;\right),\left(\;X_{2},\omega_{2}\;\right),\left(\;X_{3},\omega_{3}\;\right),\ldots ,\left(\;X_{n},\omega_{n}\;\right)\;\right\}. \end{array}$$

During the learning stage, the SVM finds parameters **W**_*i*_ = [*W*_*i*_^1^, *W*_*i*_^2^,…, *W*_*i*_^*n*^]^*T*^ and *b* to produce a decision function *d*(**X**_*i*_, **W**_*i*_, *b*):

(12) dXi,Wi,b=WiTXi+b=Wi·Xi+b=∑j=1nWijXij+b,

where **W**_*i*_, **X**_*i*_ ∈ *ℛ*^*n*^. As the training data are linearly separable no training data will satisfy the condition

(13) $$\begin{array}{c} d\left(\;X_{i},W_{i},b\;\right)=0. \end{array}$$

To control the separability, we consider the following inequalities:

(14) dXi,Wi,b≥1for  ωi=+1,dXi,Wi,b<1for  ωi=−1.

Sometime it is very difficult to find the perfect hyperplane which can separate the data, but if we transform the data into a higher dimension the data may be easily separable. To separate this kind of data, a kernel function can be introduced.

*Kernel Methods*. Assume a transformation *ϕ* such that it transforms the dataset **X**^1^ ∈ *ℛ*^*n*^ into dataset **X**^2^ ∈ *ℛ*^*m*^ where *m* > *n*. Now train the linear SVM on the dataset **X**^2^ to get a new classifier *F*_SVM_.

A kernel *ϕ* effectively computes a dot product in a higher-dimensional space *ℛ*^*m*^. For {**x**_*i*_, **x**_*j*_} ∈ *ℛ*^*N*^, *K*(**x**_*i*_, **x**_*j*_) = 〈*ϕ*(**x**_*i*_, **x**_*j*_)〉_*m*_ is an inner product of *ℛ*^*m*^, where *ϕ*(**x**) transforms **x** to *ℛ*^*m*^. Consider {**x**_*i*_, **x**_*j*_} ∈ *ℛ*^*n*^; then we can define the kernel as follows:

- Radial basis function kernel (rbf): *K*(**x**_*i*_, **x**_*j*_) = exp⁡(−*γ*|<*ϕ*(**x**_*i*_ − **x**_*j*_)>|^2^).
- Polynomial kernel (polynomial): *K*(**x**_*i*_, **x**_*j*_) = (〈*ϕ*(**x**_*i*_ · **x**_*j*_)〉 + *r*)^*d*^.
- Sigmoid kernel: *K*(**x**_*i*_, **x**_*j*_) = tanh⁡(〈*ϕ*(**x**_*i*_, **x**_*j*_)〉 + *r*).
- Linear kernel (linear): *K*(**x**_*i*_, **x**_*j*_) = 〈*ϕ*(**x**_*i*_, **x**_*j*_)〉.

The advantage of the kernel method for breast cancer image classification using an SVM was first introduced by El-Naqa et al. [156]. They classify Microcalcification clusters in mammogram images (76 images were utilized for the experiment where the total number of MCs was 1120). They utilized the SVM method along with the Gaussian kernel as well as the polynomial kernel. In 2003, Chang et al. classified a set of sonography images using SVM techniques where they consider that the image is surrounded by pickle noise [157], where the database contains 250 images. Their achieved Accuracy was 93.20%. A total of thirteen features, including shape, law, and gradient features, were utilized along with SVM and a Gaussian kernel for the mammogram image classification. They performed their operation on 193 mammogram images and achieved 83.70% sensitivity and 30.20% False Positive Rate [158]. SVM has been combined with the NN method by B. Sing et al. for ultrasound breast image classification where the database contained a total of 178 images. They performed a hybrid feature selection method to select the best features [159].

A breast ultrasound image is always very complex in nature. The Multiple Instance Learning (MIL) algorithm has been first used along with SVM for the breast image classification by [176], and their obtained Accuracy was 91.07%. The Concentric Circle BOW feature extraction method was utilized to extract the features and later the SVM method was used for breast image classification [177]. Their achieved Accuracy is 88.33% when the dimension of the features was 1000. A Bag of Features has been extracted from histopathological images (using SIFT and DCT) and using SVM for classification by Mhala and Bhandari [178]. The experiment is performed on a database which contains 361 images, where 119 images are normal, 102 images are ductal carcinoma in situ, and the rest of the images are invasive carcinoma. Their experiment achieved 100.00% classification Accuracy for ductal carcinoma in situ, 98.88% classification Accuracy for invasive carcinoma, and 100.00% classification Accuracy for normal image classification. A mammogram (DDSM) image database has been classified by Hiba et al. [179] by SVM along with the Bag of Feature method. Firstly the authors extract LBP and quantize the binary pattern information for feature extraction. Their obtained Accuracy was 91.25%.

Along with the above-mentioned work different breast image databases have been analyzed and classified using SVM. We have summarized some of the work related to SVM in Tables 14, 15, and 16.

#### 3.1.5. Bayesian

A Bayesian classifier is a statistical method based on Bayes theorem. This method does not follow any explicit decision rule; however it depends on estimating probabilities. The Naive Bayes method can be considered one of the earlier Bayesian learning algorithms.

The Naive Bayes (NB) method works on the basis of the Bayes formula, where each of the features is considered statistically independent. Consider a dataset with *m* samples, with each sample containing a feature vector **x**^**k**^ with *n* features [180] and belonging to a particular class *c*_*k*_. According to the NB formula, the probability of the particular class *c*_*k*_ with the conditional vector **x**^**k**^ is represented as

(15) $$\begin{array}{c} P\left(\;c_{k}\;|\;x^{k}\;\right)=\frac{P\left(x^{k}\;|\;c_{k}\right)P\left(c_{k}\right)}{P\left(x^{k}\right)}. \end{array}$$

Applying the chain rule

(16) $$\begin{array}{c} P\left(\;x_{1}^{k},x_{2}^{k},x_{3}^{k},\ldots ,x_{n}^{k}\;|\;c_{k}\;\right)=\overset{n}{\underset{i=1}{\prod }}P\left(\;x_{i}^{k}\;|\;c_{k}\;\right). \end{array}$$

The NB theorem considers all the features independently which can be represented as

(17) $$\begin{array}{c} \bar{c}=arg\underset{k\in 1\cdots m}{max}P\left(\;c_{k}\;\right)\overset{n}{\underset{i=1}{\prod }}P\left(\;x_{i}^{k}\;|\;c_{k}\;\right). \end{array}$$

The NB method is very easy to construct and very first to predict the data. This method can also utilize the kernel method. However, for a large dataset and continuous data, this method has very poor performance. NB can be classified into the following subclasses:

- Gaussian Naive Bayes
- Multinomial Naive Bayes
- Bernoulli Naive Bayes.

One of the constraints of the NB classifier is that it considers that all the features are conditionally independent. A Bayesian Network is another Bayesian classifier which can overcome this constraint [181, 182]. The literature shows that the Bayesian classifier method is not utilized much for breast image classification. In 2003 Butler et al. used NB classifier for X-ray breast image classification [183]. They extracted features from the low-level pixels. For all feature combinations they obtained more than 90.00% Accuracy. Bayesian structural learning has been utilized for a breast lesion classifier by Fischer et al. [184]. Soria et al. [185] classify a breast cancer dataset utilizing C4.5, multilayered perceptron, and the NB algorithm using WEKA software [186]. They conclude that the NB method gives better performance than the other two methods in that particular case. They also compared their results with the Bayes classifier output. Some other research on the Bayes classifier and breast image classification has been summarized in Tables 17 and 18.

### 3.2. Performance Based on Unsupervised Learning

This learning algorithm does not require any prior knowledge about the target. The main goal of the unsupervised learning is to find the hidden structure and relations between the different data [187] and distribute the data into different clusters. Basically clustering is a statistical process where a set of data points is partitioned into a set of groups, known as a cluster. The *K*-means algorithm is a clustering algorithm proposed by [188]. Interestingly, unsupervised learning can be utilized as preprocessing step too.

(i) In the *K*-means algorithm, firstly assign *K* centroid points. Suppose that we have *n* feature points *x*_*i*_ where *i* ∈ {1,…, *n*}. The objective of the *K*-means algorithm is to find positions *μ*_*i*_, where *i* ∈ 1,…, *K* that minimize the data points to the cluster by solving
  (18) $$\begin{array}{c} arg\;\underset{x\in c_{i}}{min}\overset{K}{\underset{i=1}{\sum }}\;\underset{x\in c_{i}}{\sum }d\left(\;x,\mu_{i}\;\right)=arg\;\underset{x\in c_{i}}{min}\overset{K}{\underset{i=1}{\sum }}\;\underset{x\in c_{i}}{\sum }{\left\|\;x-\mu_{i}\;\right\|}^{2}. \end{array}$$
(ii) Self-Organizing Map (SOM): SOM is another popular unsupervised classifier, proposed by Kohonen et al. [189–191]. The main idea of the SOM method is to reduce the dimension of the data and represent those dimensionally reduced data by a map architecture, which provides more visual information.
(iii) Fuzzy *C*-Means Clustering (FCM): the FCM algorithm cluster databased on the value of a membership function is proposed by [192] and improved by Bezdek [193].

The history of using unsupervised learning for breast image classification is a long one. In 2000, Cahoon et al. [194] classified mammogram breast images (DDSM database) in an unsupervised manner, utilizing the *K*-NN clustering and Fuzzy *C*-Means (FCM) methods. Chen et al. classified a set of breast images into benign and malignant classes [164]. They utilized a SOM procedure to perform this classification operation. They collected 24 autocorrelation textural features and used a 10-fold validation method. Markey et al. utilized the SOM method for BIRADS image classification of 4435 samples [195]. Tables 19 and 20 summarize the breast image classification performance based on *K*-means algorithm and SOM method.

### 3.3. Performance Based on Semisupervisor

The working principle of semisupervised learning lies in between supervised and unsupervised learning. For the semisupervised learning a few input data have an associated target and large amounts of data are not labeled [196]. It is always very difficult to collect the labeled data. Few data such as speech or information scratched from the web are difficult to label. To classify this kind of data semisupervised learning is very efficient. However lately this method has been utilized for the brats image classification too. Semisupervised learning can be classified as

- Graph Based (GB)
- Semisupervised Support Vector Machine
- Human Semisupervised Learning.

To the best of our knowledge, Li and Yuen have utilized GB semisupervised learning for biomedical image classification [197]. The kernel trick is applied along with the semisupervised learning method for breast image classification by Li et al. [198]. They performed their experiments on the Wisconsin Prognostic Breast Cancer (WPBC) dataset for the breast image classification. Ngadi et al. utilized both the SKDA (Supervised Kernel-Based Deterministic Annealing) and NSVC methods for mammographic image classification [199]. They performed their experiments on 961 images, where 53.60% of the images were benign and the rest of the images are malignant. Among the other utilized features they utilized BI-RADS descriptors as features. When they utilized the NSVC method they also utilized RBF, polynomial, and linear kernel. They found that the best Accuracy of 99.27% was achieved when they utilized linear kernels. Few studies have performed the breast image classification by semisupervised learning, as summarized in Tables 21 and 22.

## 4. Conclusion

Breast cancer is a serious threat to women throughout the world and is responsible for increasing the female mortality rate. The improvement of the current situation with breast cancer is a big concern and can be achieved by proper investigation, diagnosis, and appropriate patient and clinical management. Identification of breast cancer in the earlier stages and a regular check of the cancer can save many lives. The status of cancer changes with time, as the appearance, distribution, and structural geometry of the cells are changing on a particular time basis because of the chemical changes which are always going on inside the cell. The changing structure of cells can be detected by analysing biomedical images which can be obtained by mammogram, MRI, and so forth techniques. However these images are complex in nature and require expert knowledge to perfectly analyze malignancy. Due to the nontrivial nature of the images the physician sometimes makes a decision which might contradict others. However computer-aided-diagnosis techniques emphasising the machine learning can glean a significant amount of information from the images and provide a decision based on the gained information, such as cancer identification, by classifying the images.

The contribution of machine learning techniques to image classification is a long story. Using some advanced engineering techniques with some modifications, the existing machine learning based image classification techniques have been used for biomedical image classification, specially for breast image classification and segmentation. A few branches of the machine learning based image classifier are available such as Deep Neural Network, Logic Based, and SVM. Except for deep-learning, a machine learning-based classifier largely depends on handcrafted feature extraction techniques such as statistical and structural information that depend on various mathematical formulations and theorize where they gain object-specific information. They are further utilized as an input for an image classifier such as SVM and Logic Based, for the image classification.

This investigation finds that most of the conventional classifiers depend on prerequisite local feature extraction. The nature of cancer is always changing, so the dependencies on a set of local features will not provide good results on a new dataset. However the state-of-the art Deep Neural Networks, specially CNN, have recently advanced biomedical image classification due to the Global Feature extraction capabilities. As the core of the CNN model is the kernel, which gives this model the luxury of working with the Global Features, these globally extracted features allow the CNN model to extract more hidden structure from the images. This allows some exceptional results for breast cancer image classification. As the CNN model is based on the Global Features, this kind of classifier model should be easy to adapt to a new dataset.

This paper also finds that the malignancy information is concentrated in the particular area defined as ROI. Utilizing only the ROI portions, information gathered from the segmented part of the data can improve the performance substantially. The recent development of the Deep Neural Network can also be utilized for finding the ROI and segmenting the data, which can be further utilized for the image classification.

For breast cancer patient care, the machine learning techniques and tools have been a tremendous success so far, and this success has gained an extra impetus with the involvement of deep-learning techniques. However the main difficulty of handling the current deep-learning based machine learning classifier is its computational complexity, which is much higher than for the traditional method. The current research is focused on the development of the light DNN model so that both the computational and timing complexities can be reduced. Another difficulty of using the DNN based cancer image classifier is that it requires a large amount of training data. However the reinforcement of learning techniques and data augmentation has been largely adapted with the current CNN model, which can provide reliable outcomes. Our research finds that the current trend of machine learning is largely towards deep-learning techniques. Among a few other implications, the appropriate tools for designing the overall deep-learning model was the initial obligation for utilizing deep-learning based machine learning techniques. However some reliable software has been introduced which can be utilized for breast image classification. Initially it was difficult to implement a DNN based architecture in simpler devices; however due to cloud-computer based Artificial Intelligence techniques this issue has been overcome and DNN has already been integrated with electronic devices such as mobile phones. In future combining the DNN network with the other learning techniques can provide more-positive predictions about breast cancer.

Due to the tremendous concern about breast cancer, many research contributions have been published so far. It is quite difficult to summarize all the research work related to breast cancer image classification based on machine learning techniques in a single research article. However this paper has attempted to provide a holistic approach to the breast cancer image classification procedure which summarizes the available breast dataset, generalized image classification techniques, feature extraction and reduction techniques, performance measuring criteria, and state-of-the-art findings.

In a nutshell, the involvement of machine learning for breast image classification allows doctors and physicians to take a second opinion, and it provides satisfaction to and raises the confidence level of the patient. There is also a scarcity of expert people who can provide the appropriate opinion about the disease. Sometimes the patient might need to spend a long time waiting due to the lack of expert people. In this particular scenario the machine learning based diagnostic system can help the patient to receive the timely feedback about the disease which can improve the patient-management scenario.

## Figures and Tables

**Table 1:** Available breast image database for biomedical investigation.

Database	Number of images	Database size (GB)	Image capture technique	Image type	Total patients
MIAS	322	2.3	Mammogram		161
DDSM			Mammogram		2620
CBIS-DDSm	4067	70.5	MG	DICOM	237
ISPY1	386,528	76.2	MR, SEG		237
Breast-MRI-NACT-Pilot	99,058	19.5	MRI		64
QIN-Breast	100835	11.286	PET/CT, MR	DICOM	67
Mouse-Mammary	23487	8.6	MRI	DICOM	32
TCGA-BRCA	230167	88.1	MR, MG	DICOM	139
QIN Breast DCE-MRI	76328	15.8	CT	DICOM	10
BREAST-DIAGNOSIS	105050	60.8	MRI/PET/CT	DICOM	88
RIDER Breast MRI	1500	.401	MR	DICOM	5
BCDR			Mammogram		1734
TCGA-BRCA		53.92 (TB)	Histopathology		1098
BreakHis	7909		Histopathology		82
Inbreast	419		Mammogram		115

**Table 2:** Feature descriptor.

Feature category	Feature description
Texture	Haralick texture features [7]
	(1) Angular Second Moment (ASM), (2) Contrast, (3) correlation, (4) Sum of Squares of Variances (SSoV), (5) Inverse of Difference (IoD), (6) Sum of Average (SoA), (7) Sum of Variances (SoV), (8) Sum of Entropy (SoE), (9) Entropy, (10) Difference of Variance (DoV), (11) Difference of Entropy (DoE), (12) Gray-Level Concurrence Matrix (GLCM).
	Tamura features [8]
	(1) Coarseness, (2) Contrast, (3) directionality, (4) line-likeness, (5) roughness, (6) regularity.
	Global texture descriptor
	(1) Fractal dimension (FD), (2) Coarseness, (3) Entropy, (4) Spatial Gray-Level Statistics (SGLS), (5) Circular Moran Autocorrelation Function (CMAF).

Detector	Single scale detector
	(1) Moravec's Detector (MD) [9], (2) Harris Detector (HD) [10], (3) Smallest Univalue Segment Assimilating Nucleus (SUSAN) [11], (4) Features from Accelerated Segment Test (FAST) [12, 13], (5) Hessian Blob Detector (HBD) [14, 15].
	Multiscale detector [8]
	(1) Laplacian of Gaussian (LoG) [9, 16], (2) Difference of Gaussian (DoG) Contrast [17] (3) Harris Laplace (HL), (4) Hessian Laplace (HeL), (5) Gabor-Wavelet Detector (GWD) [18].

Strutural	(1) Area, (2) bounding box, (3) centroid, (4) Convex Hull (CH), (5) eccentricity, (6) Convex Image (CI), (7) compactness, (8) Aspect Ratio (AR), (9) moments, (10) extent, (11) extrema, (12) Major Axis Length (MaAL), (13) Minor Axis Length (MiAL), (14) Maximum Intensity (MaI), (15) Minimum Intensity (MiI), (16) Mean Intensity (MI), (17) orientation, (18) solidity.

**Table 3:** Feature descriptor.

Feature category	Feature description
Statistical	(1) Mean, (2) Median, (3) Standard Deviation, (4) Skewness, (5) Kurtosis, (6) Range

Descriptor	(1) Scale Invariant Feature Transform (SIFT) [17, 19], (2) Gradient Location-Orientation Histogram (GLOH) [20], (3) Speeded-Up Robust Features Descriptor (SURF) [21–23], (4) Local Binary Pattern (LBP), [24–27], (5) Binary Robust Independent Elementary Features (BRIEF) [28], (6) Weber Local Descriptor (WLD) [29, 30] (7) Back Ground Local Binary Pattern (BGLBP) [31], (8) Center-Symmetric Local Binary Pattern (CS-LBP), [32] (9) Second-Order Center-Symmetric Local Derivative Pattern (CS-LBP) [33], (10) Center-Symmetric Scale Invariant Local Ternary Patterns (CS-SILTP) [34], (11) Extended LBP or Circular LBP (E-LBP) [35], (12) Opponent Color Local Binary Pattern (OC-LBP), [36] (13) Original LBP(O-LBP) [25], (14) Spatial Extended Center-Symmetric Local Binary Pattern (SCS-LBP) [37], (15) Scale Invariant Local Ternary Pattern (SI-LTP) [38], (16) Variance-Based LBP (VAR-LBP) [24], (17) eXtended Center-Symmetric Local Binary Pattern (XCS-LBP), (18) Average Local Binary Pattern (ALBP), (19) Block Based Local Binary Pattern (BBLBP) [39],

BI-RADS [40]	(1) Margin Integrality (MarI), (2) Margin Ambiguity (MarA), (3) Echo Pattern Posterior Feature (EPPF), (4) Calcification in Mass (CM), (5) Architectural Distortion (AD), (6) Edema, (7) Eymph Nodes Axillary (ENA) (8) Ducts Changes (DC), (9) Skin Thickening (ST), (10) Postsurgical Fluid Collection (PSFC), (11) Skin Retraction (SR1), (12) Fat Necrosis (FN), (13) Lump Nodes Intramammary (LNI).

**Table 4:** A simplified hierarchy of classification.

Learning technique	Algorithm		
Supervised	Conventional	(a) Logic based	(1) ID3, (2) C4.5, (3) bagging,
			(4) random trees, (5) Random Forest,
			(6) boosting, (7) advanced boosting,
			(8) Extreme Boosting (XGBoosting).
		(b) Bayesian	(1) Naive Bayes
			(2) Bayesian Network
		(c) Conventional Neural Network	
		(d) Support Vector Machine	
	DNN-based	(a) Convolutional Neural Network (CNN),	
		(b) Deep Belief Network (DBN),	
		(c) Generative Adversial Network (GAN).	

Unsupervised	Conventional	(a) *k*-Means Clustering	
		(b) Self-Organizing Map (SOP)	
		(c) Fuzzy *C*-Means Clustering (FCM)	
	DNN-based	(a) Deep Belief Network (DBN)	

Semisupervised	Conventional	(a) Self-training	
		(b) Graph Based	
		(c) S3V3	
		(d) Multiview	
		(e) Generative model	

**Table 5:** Neural Network for breast image classification.

Reference	Descriptor	Image type	Number of images	Key findings
Rajakeerthana et al. [42]	(1) GLCM, GLDM, SRDM, NGLCM, GLRM	Mammogram	322	(1) The classifier achieved 99.20% Accuracy.

Lessa and Marengoni [43]	(1) Mean, Median, Standard Deviation, Skewness, Kurtosis, Entropy, Range	Thermographic	94	(1) Achieved Sensitivity, Specificity, and Accuracy are 87.00%, 83.00%, and 85.00%, respectively.

Wan et al. [44]	(1) ALBP (2) BBLBP	OCM	46	(1) Achieved Sensitivity and Specificity are 100% and 85.20%. respectively.
				(2) ROC value obtained 0.959.

Chen et al. [40]	(1) 19 BI-RADS features have been used	Ultrasound	238	(1) Chi squared method has been utilized for the feature selection.
				(2) Achieved Accuracy, Sensitivity, and Specificity are 96.10%, 96.70%, and 95.70%, respectively.

de Lima et al. [45]	(1) Total 416 features have been used	Mammogram	355	(1) Multiresolution wavelet and Zernike moment have been utilized for the feature extraction.

Abirami et al. [46]	(1) 12 statistical measures such as Mean, Median, and Max have been utilized as the features	Mammogram	322	(1) Wavelet transform has been utilized for the feature extraction.
				(2) The achieved Accuracy, Sensitivity, and Specificity are 95.50%, 95.00%, and 96.00%, respectively.

El Atlas et al. [47]	(1) 13 morphological features have been utilized	Mammogram	410	(1) Firstly the edge information has been utilized for the mass segmentation and then the morphological features were extracted.
				(2) Achieved best Accuracy is 97.5%.

**Table 6:** Neural Network for breast image classification.

Reference	Descriptor	Image type	Number of images	Key findings
Alharbi et al. [48]	(1) 49 features have been utilized.	Mammogram	1100	(1) Five feature selection methods: Fisher score, Minimum Redundancy-Maximum Relevance, Relief-f, Sequential Forward Feature Selection, and Genetic Algorithm have been used.
				(2) Achieved Accuracy, Sensitivity, and specificity are 94.20%, 98.36%, and 99.27%, respectively

Peng et al. [49]	(1) Haralick and Tamura features have been utilized	Mammogram	322	(1) Feature reduction has been performed by Rough-Set theory and selected 5 prioritized features.
				(2) The best Accuracy, Sensitivity, and Specificity achieved were 96.00%, 98.60%, and 89.30%

Jalalian et al. [50]	(1) GLCM	Mammogram		(1) The obtained classifier Accuracy, Sensitivity, and Specificity are 95.20%, 92.40%, and 98.00%, respectively.
	(2) Compactness			

Li et al. [51]	(1) Four feature vectors have been calculated	Mammogram	322	(1) 2D contour of breast mass in mammography has been converted into 1D signature.
				(2) NN techniques achieved Accuracy is 99.60% when RMS slope is utilized.

Chen et al. [52]	(1) Autocorrelation features	Ultrasound	242	(1) The overall achieved Accuracy, Sensitivity, and Specificity are 95.00%, 98.00%, and 93%, respectively.

Chen et al. [53]	(1) Autocorrelation features	Ultrasound	1020	(1) The obtained ROC area is 0.9840 ± 0.0072.

**Table 7:** Neural Network for breast image classification.

Reference	Descriptor	Image type	Number of images	Key findings
Chen et al. [61]	(1) Variance Contrast of Wavelet Coefficient	Ultrasound	242	(1) The achieved ROC curve 0.9396 ± 0.0183
	(2) Autocorrelation of Wavelet Coefficient			

Silva et al. [62]	(1) 22 different morphological features such as convexity and lobulation have been utilized	Ultrasound	—	(1) The best obtained Accuracy and ROC curve are 96.98% and 0.98, respectively

Saritas [63]	(1) Age of patient, (2) mass shape, (3) mass border, (4) Mass density, (5) BIRADS	Mammogram	—	(1) Disease prediction rate is 90.5%
				(2) Neural Network utilized 5 neurons in input layers and one hidden layer.

López-Meléndez et al. [64]	(1) Area, perimeter, etc. have been utilized	Mammogram	322	(1) The achieved Sensitivity and Specificity are 96.29% and 99.00%, respectively.

**Table 8:** Available software for deep learning analysis.

Software	Interface and backend	Provider
Caffe [65, 66]	Python, MATLAB, C++	Berkeley Vision and Learning Centre, University of California, Berkeley
Torch [67]	C, LuaJIT	
MatConvNet [68, 69]	MATLAB, C	Visual Geometry Group, Department of Engineering, University of Oxford
Theano [70, 71]	Python	Montreal Institute for Learning Algorithms
		University of Montreal
TensorFlows [72]	C++, Python	Google
CNTK [73]	C++	Microsoft
Keras [74]	Theano, Tensor Flow	MIT
dl4j [75]	Java	Skymind Engineering
DeeBNET [76, 77]	MATLAB	Information Technology Department, Amirkabir University of Technology

**Table 9:** Convolutional Neural Network.

Reference	Descriptor	Image type	Number of images	Key findings
Wu et al. [78]	(1) Global Features	Mammogram	40	(1) Achieved Sensitivity 75.00% and Specificity 75.00%.

Sahiner et al. [79]	(1) Global Features	Mammogram	168	(1) The achieved ROC score is 0.87.

Lo et al. [80]	(1) Density, size, Shape, Margin	Mammogram	144	(1) The achieved ROC curve is 0.89.

Fonseca et al. [81]	(1) Global Features	Mammogram	—	(1) Breast density classification has been performed utilizing HT-L3 convolution.
				(2) Average achieved obtained Kappa value is 0.58.

Arevalo et al. [82]	(1) Global Features	Mammogram	736	(1) The achieved ROC curve is 0.826.

Su et al. [83]	(1) Global Features	Mammogram	92	(1) Fast Scanning CNN (fCNN) method has been utilized to reduce the information loss.
				(2) The average Precision, Recall, and *F*1 score are 91.00%, 82.00%, and 0.85, respectively.

Sharma and Preet [84]	(1) GLCM, GLDM Geometrical	Mammogram	40	(1) The best Accuracy achieved is 75.23% and 72.34%, respectively, for fatty and dense tissue classification.

Spanhol et al. [6]	(1) Global Features	Histopathology	7909	(1) The best Accuracy achieved 89 ± 6.6%.

Rezaeilouyeh et al. [85]	(1) Local and Global Features	Histopathology	—	(1) Shearlet transform has been utilized for extracting local features.
				(2) When they utilize RGB image along with magnitude of Shearlet transform together, the Achieved Sensitivity, Specificity, and Accuracy were 84.00 ± 1.00%, 91.00 ± 2.00%, and 84.00 ± 4.00%; when they utilize RGB image along with both the phase and magnitude of Shearlet transform together, the achieved Sensitivity, Specificity, and Accuracy were 89.00 ± 1.00%, 94.00 ± 1.00%, and 88.00 ± 5.00%.

**Table 10:** Convolutional Neural Network.

Reference	Descriptor	Image type	Number of images	Key findings
Albayrak and Bilgin [86]	(1) Global Features	Histopathology	100	(1) Cluster-based segmentation has been performed to find out the cellular structure.
				(2) Blob analysis has been performed on the segmented images.
				(3) To reduce the high dimensionality, Principal Component Analysis (PCA) and Linear Discriminant Analysis (LDA) methods have been utilized.
				(4) Before the dimensionality reduction the Precision, Recall, and *F*-score values were 97.20%, 66.00%, and 0.78%, respectively, but when the dimensionality reduction method was utilized the Precision, Recall, and *F*-score values were 100.00%, 94.00%, and 0.96%, respectively
				(5) The best average Accuracy is 73.00% (without dimensionality reduction) and 96.8% (with dimensionality reduction).

Jiao et al. [87]	(1) Global and Local Features.	Mammogram	—	(1) They performed their experiments on the DDSM database.
				(2) Total required parameter is 5.8 × 10^7^ and time for the per image processing is 1.10 ms.
				(3) The best classification achieved is 96.70%; however they show that when they utilize the VGG model the Accuracy was 97.00% which is slightly better than their model.
				However in terms of memory size and time per image processing their model gives better performance than the VGG model.

Zejmo et al. [88]	(1) Global Features	Cytology	40	(1) GoogleNet and AlexNet models have been utilized.
				(2) The best Accuracy obtained when they utilized GoogleNet model was 83.00%.

**Table 11:** Convolutional Neural Network.

Reference	Descriptor	Image type	Number of images	Key findings
Jiang et al. [89]	(1) Global Features	Mammogram	—	(1) Image preprocessing was performed to enhance tissue characteristics.
				(2) Transfer learning was performed and obtained AUC was 0.88 whereas when the system learned from scratch, the best ROC is 0.82.

Suzuki et al. [90]	(1) Global Features	Mammogram	198	(1) The achieved sensitivity 89.90%.
				(2) Transfer learning techniques have been utilized.

Qiu et al. [91]	(1) Global Features	Mammogram	270	(1) Average achieved Accuracy is 71.40%.

Samala et al. [92]	(1) Global Features	—	92	(1) They utilized Deep Learning CNN (DLCNN) and CNN models for classification.
				(2) The AUC of CNN and DLCNN model is 0.89 and 0.93, respectively.

Sharma and Preet [84]	(1) Global Features	Mammogram	607	(1) Transfer learning and ensemble techniques utilized.
				(2) When using ensemble techniques the soft voting method has been used.
				(3) The best ROC score is 0.86.

Kooi et al. [93]	(1) Global and Local features	Mammogram	44090	(1) Transfer learning method utilized (VGG model).

Geras et al. [94]	(1) Global Features	Mammogram	102800	(1) They investigated the relation of the Accuracy with the database size and image size.

Arevalo et al. [82]	(1) Global Features	Mammogram	736	(1) The best ROC value was 0.822.

**Table 12:** Logic Based.

Reference	Descriptor	Image type	Number of images	Key findings
Beura et al. [95]	(1) Two-dimensional discrete orthonormal *S*-transform has been used for the feature extraction	Mammogram	—	(1) Achieved Accuracy and AUC values on MIAS database are 98.3%, 0.9985. (2) Achieved Accuracy and AUC values on DDSM database are 98.8%, 0.9992.

Diz et al. [96]	(1) GLCM	Mammogram	410	(1) Their achieved Accuracy value is 76.60%
	(2) GLRLM			(2) Mean false positive value is 81.00%.

Zhang et al. [97]	(1) 133 features (mass based and content based)	Mammogram	400	(1) Computer model has been created which is able to find a location that was not detected by trainee.

Ahmad and Yusoff [98]	(1) Nine features selected	Biopsy	700	(1) Achieved Sensitivity, Specificity, and Accuracy are 75.00%, 70.00%, and 72.00%, respectively.

Paul et al. [99]	(1) Harlick texture feature	Histopathological	50	(1) Their achieved Recall and Precision are 81.13% and 83.50%.

Chen et al. [100]	(1) Dual-tree complex wavelet transform (DT-CWT) has been used for the feature extraction.	Mammogram	—	(1) Achieved Received Operating Curve (ROC) 0.764.

Zhang et al. [101]	(1) Curvelet Transform (2) GLCM (3) CLBP	Histopathological	50	(1) Random Subspace Ensemble (RSE) utilized. (2) Their achieved classification Accuracy is 95.22% where the previous Accuracy on this same database was 93.40%.

**Table 13:** Logic Based.

Reference	Descriptor	Image type	Number of images	Key findings
Angayarkanni and Kamal [102]	(1) GLCM	Mammogram	322	(1) The Achieved Sensitivity and Accuracy are 93.40% and 99.50%, respectively.

Wang et al. [103]	(1) Horizontal Weighted Sum (2) Vertical Weighted Sum (3) Diagonal Weighted Sum (4) Grid Weighted Sum.	Mammogram	322	(1) Surrounding Region Dependence Method (SRDM) utilized for region detection. (2) Achieved True Positive Rate 90.00% and False Positive Rate 88.80%.

Tambasco Bruno et al. [104]	(1) Curvelet Transform (2) LBP	Mammogram Histopathological	—	(1) ANOVA method utilized for feature prioritization. (2) When they use RF algorithm on Mammogram (DDSM) dataset, obtained Accuracy and ROC are 79.00% and 0.89.

Muramatsu et al. [105]	(1) Radial Local Ternary Pattern (RLTP)	Mammogram	376	(1) Textural features have been extracted from the regions of interest (ROIs) using RLTP. (2) They claimed that the RLTP feature provides better performance than the rotation invariant patterns.

Dong et al. [106]	(1) NRL margin gradient (2) Gray-level histogram (3) Pixel value fluctuation	Mammogram	—	(1) Chain code utilized for extraction of regions of interest (ROIs). (2) Rough-Set method utilized to enhance the ROIs. (3) Their achieved ROC value is 0.947 and obtained Matthews Correlation (MCC) is 0.8652.

Piantadosi et al. [107]	(1) Local Binary Pattern-Three Orthogonal Projections (LBP-TOP)	Mammogram	—	(1) Their achieved Accuracy, Sensitivity, and Specificity values are 84.60%, 80.00%, and 90.90%.

**Table 14:** SVM for breast image classification (Page-1).

Reference	Descriptor	Image type	Number of images	Key findings
Malik et al. [108]	(1) Speed of sound (2) Attenuation image vector (3) Reflection image vector	QTUS	—	(1) Glands, fat, skin, and connective tissue have been classified. (2) Both linear and nonlinear SVM classifier have been utilized. (3) Their experiment obtained 85.20% Accuracy.

Chang et al. [109]	(1) Textural features such as (i) Autocorrelation Coefficient (ii) Autocovariance Coefficient	Ultrasound	250	(1) Benign and malignant images have been classified. (2) Accuracy, Sensitivity, Specificity, positive predictive values, and negative predictive value are 85.60%, 95.45%, 77.86%, 77.21%, and 95.61%, respectively.

Akbay et al. [110]	(1) 52 features have been extracted	Mammogram	—	(1) Microcalcification (MC) Classification Accuracy 94.00%

Levman et al. [111]	(1) Relative Signal Intensities (2) Derivative of Signal Intensities (3) Relative Signal Intensities and their derivatives in one vector (4) (i) Maximum of signal intensity enhancement; (ii) time of maximum enhancement; (iii) time of maximum washout	MRI	76	(1) Benign and malignant lesions are investigated. (2) Linear kernel, a polynomial kernel, and a radial basis function kernel utilized along with the SVM method for the breast image classification.

de Oliveira Martins et al. [112]	(1) Ripley's *K* function	Mammogram	390	(1) Benign and malignant image classification. (2) The achieved Accuracy, Sensitivity, and Specificity are 94.94%, 92.86%, and 93.33%, respectively.

**Table 15:** SVM for breast image classification.

Reference	Descriptor	Image type	Number of images	Key findings
Zhang et al. [122]	(1) Fractional Fourier transform information utilized as features	Mammogram	200	(1) They selected ROI for avoiding redundant complexity. (2) When SVM and Principal Component Analysis were used together the achieved Accuracy, Sensitivity and Specificity are 92.16 ± 3.60%, 92.10 ± 2.75% and 92.22 ± 4.16% respectively.

Shirazi and Rashedi [123]	(1) GLCM	Ultrasound	322	(1) ROI extracted for reducing redundant complexity. (2) SVM and Mixed Gravitational Search Algorithm (MGSA) used together for feature reduction. (3) The achieved Accuracy 86.00%; however SVM with MGSA method achieved 93.10% Accuracy.

Sewak et al. [124]	(1) Radius, perimeter, area, compactness, smoothness, concavity, concave points, symmetry, fractal dimension, and texture of nuclei calculated	Biopsies	569	(1) Achieved Accuracy, Sensitivity, and Specificity are 99.29%, 100.00%, and 98.11%, respectively.

Dheeba and Tamil Selvi [125]	(1) The laws texture features utilized	Mammogram	322	(1) The achieved Accuracy is 86.10%.

**Table 16:** SVM for breast image classification.

Reference	Descriptor	Image type	Number of images	Key findings
Taheri et al. [126]	(1) Intensity information (2) Value of detected corner (3) Energy	Mammogram	600	(1) Classified images into normal and abnormal images. (2) Removing unwanted objects from the images for reducing the redundancy and computational complexity. (3) Achieved Precision and Recall rates are 96.80% and 92.5%, respectively.

Tan et al. [127]	(1) Shape, fat, presence of calcification texture, spiculation, Contrast, Isodensity type features selected (2) Total number of features 181	Mammogram	1200	(1) Features have been selected from the region of interest. (2) They utilized the radial basis function (RBF) for their analysis. (3) The Sequential Forward Floating Selection (SFFS) method utilized for the feature selection. (4) The area under the receiver operating characteristic curve was (AUC) = 0.805 ± 0.012.

Kavitha and Thyagharajan [128]	(1) Histogram of the intensity has been used as a statistical feature. (2) 2D Gabor filter utilized for the textural feature extraction (3) Clinical features extracted from the database directly	Mammogram	322	(1) When using SVM with the linear kernel the obtained Accuracy, Sensitivity, and Specificity are 98%, 100%, and 96%, respectively. (2) When using weighted feature SVM with weights the obtained Accuracy, Sensitivity, and Specificity are 90%, 100% and 75%, respectively.

**Table 17:** Bayesian classifier.

Reference	Descriptor	Image type	Number of images	Key findings
Kendall and Flynn [129]	(1) Features extracted using DCT method.	Mammogram		(1) Bayesian classifier obtained 100.00% sensitivity with 64.00% specificity.

Oleksyuk et al. [130]		—	—	(1) Bayesian method obtained 86.00% with 80.00% specificity.

Burling-Claridge et al. [131]	(1) Statistical and LBP features extracted.	Mammogram	322/410	(1) Bayesian method obtained 67.07 ± 0.73% and 67.61 ± 0.83% Accuracy on MIAS and Inbreast image datasets (using statistical features). (2) Bayesian method obtained 62.86 ± 0.70% and 51.99 ± 1.28% Accuracy on MIAS and Inbreast image datasets (using LBP).

Raghavendra et al. [132]	(1) Gabor wavelet transform utilized for feature extraction.	Mammogram	690	(1) Locality Sensitive Discriminant Analysis (LSDA) for the data reduction. (2) NB obtained 84.34% Accuracy and 83.69% Sensitivity with 90.86% Specificity.

Pérez et al. [133]	(1) 23 features utilized.	Mammogram	—	(1) UFilter feature selection methods utilized and its efficiency verified by Wilcoxon statistical test.

Rashmi et al. [134]	(1) 10 features utilized.	—	—	(1) Benign and malignant tumors have been classified.

Gatuha and Jiang [135]	(1) 10 features utilized.	—	—	(1) They built an android based benign and malignant tumor classifier. (2) Their obtained Accuracy is 96.4%

**Table 18:** Bayesian classifier.

Reference	Descriptor	Image type	Number of images	Key findings
Benndorf et al. [136]	(1) BI-RADS features utilized.	—	2766	(1) For the training data the AUC value is 0.959 for the inclusive model, whereas AUC value is 0.910 for the descriptor model.

Rodríguez-López and Cruz-Barbosa [137]	(1) Eight image feature nodes utilized.	—	—	(1) NB model obtained 79.00% Accuracy, 80.00% Sensitivity.

Nugroho et al. [138]	(1) Eight image feature nodes utilized.	Mammogram	—	(1) Naive Bayes model along with SMO; obtained ROC value is 0.903. (2) Bayesian Network model along with SMO; obtained Accuracy was 83.68%.

Rodríguez-López and Cruz-Barbosa [139]	(1) Eight image features have been utilized.	—	231	(1) Bayesian Network model obtained 82.00% Accuracy, 80.00% Sensitivity, and 83.00% Specificity when they utilized only three features.

Shivakumari et al. [140]		—	231	(1) Analyze the Ljubljana breast image dataset. (2) NB algorithm along with feature ranking techniques; the best achieved Accuracy was 81.46%.

Rodríguez-López and Cruz-Barbosa [141]	(1) Seven different clinical features extracted.	Mammogram	690	(1) Obtained Accuracy, Sensitivity, and Specificity are 82.00%, 80.00%, and 83.00%, respectively.

**Table 19:** *K*-means Cluster Algorithm and Self-Organizing Map for breast image classification.

Reference	Descriptor	Image type	Number of images	Key findings
Moftah et al. [142]	(1) Intensity distribution used as feature.	MRI	—	(1) Three types of evaluation measures performed: (a) Accuracy, (b) feature based, (c) shape based measure. (2) This can classify the data as well as identify the target. (3) The obtained best Accuracy of the segmented ROI is 90.83%

Lee et al. [143]	(1) 1734 signal patterns.	MRI	322	(1) Available signal patterns have been classified into 10 classes.

Dalmiya et al. [144]	(1) Discrete Wavelet Transform.	Mammogram	—	(1) Cancer tumor masses have been segmented.

Elmoufidi et al. [145]	(1) Local Binary Pattern.	Mammogram	322	(1) Image enhancing. (2) Generation of number of clusters (3) Detection of regions of interest. (4) Mean detection of regions of interest is 85.00%.

Samundeeswari et al. [146]		Ultrasound	—	(1) Utilizing ant colony and regularization parameters. (2) This method obtained 96.00% similarity between segmented and reference tumors.

Rezaee [147]	Discrete Wavelet Transform.	Mammogram	120	(1) Early detection of tumors from the breast image. (2) Tumor detection Accuracy 92.32%, Sensitivity 90.24%.

Chandra et al. [148]	(1) Gray intensity values.	Mammogram	—	(1) Mammogram image has been clustered using SOM along with the Quadratic Neural Network.

**Table 20:** *K*-means Cluster Algorithm and Self-Organizing Map for breast image classification.

Reference	Descriptor	Image Type	No. of Images	Key Findings
Lashkari and Firouzmand [160]		Thermogram	23	(1) Both FCM method and Adaboost method utilized separately to classify images. (2) For the classification purposes selected 23 features and also select the best features using feature selection algorithm. When they used the FCM method, the obtained Mean Accuracy was 75.00% whereas the Adaboost method Accuracy was 88.00%.

Nattkemper et al. [161]		MRI	—	(1) *K*-means algorithm as well as SM method utilized.

Slazar-Licea et al. [162].		⋯	—	(1) Fuzzy *c*-means algorithm used.

Marcomini et al. [163]	(1) 24 morphological features	Ultrasound	144	(1) Minimizing noise using Wiener filter, equalized and Median filter (2) Obtained Sensitivity 100% and Specificity 78.00%.

Chen et al. [164]	(1) 24 autocorrelation texture features	Ultrasound	243	(1) Obtained ROC area 0.9357 ± 0.0152. Accuracy 85.60%, Specificity 70.80%.

Iscan et al. [165]	(1) Two-dimensional discrete cosine transform (2) 2D continuous wavelet transform	Ultrasound	—	(1) Automated threshold scheme introduce to increase the robustness of the SOM algorithm.

**Table 21:** Semisupervised algorithm for breast image classification.

Reference	Descriptor	Image type	Number of images	Key finding
Cordeiro et al. [166]	(1) Zernike moments have been used for the feature extraction.	—	685	(1) Semisupervised Fuzzy GrowCut algorithm utilized. (2) For the fatty-tissue classification this method achieved 91.28% Accuracy.

Cordeiro et al. [167]	—	Mammogram	322	(1) Semisupervised Fuzzy GrowCut as well as the Fuzzy GrowCut algorithm utilized for tumors, region segmentation.

Nawel et al. [168]	—	—	—	(1) Semisupervised Support Vector Machine (S3VM) utilized. (2) This experiment shows impressive results on the DDSM database.

Zemmal et al. [169]	—	DDSM	—	(1) Transductive semisupervised learning technique using (TSVM) utilized for classification along with different features.

Zemmal et al. [170]	—	—	200	(1) Semisupervised Support Vector Machine (S3VM) utilized with various kernels.

Zemmal et al. [171]	(1) GLCM (2) Hu moments (3) Central Moments	Mammogram	—	(1) Transductive Semisupervised learning technique used for image classification. (2) This experiment shows impressive results on DDSM database.

Peikari et al. [172]	(1) Mean, Mode, Standard Deviation, Media, Skewness, Kurtosis	Histopathological	322	(1) The Ordering Points to Identify the Clustering Structure (OPTICS) method utilized for image classification [173].

**Table 22:** Semisupervised algorithm for breast image classification.

Reference	Descriptor	Image type	Number of images	Key findings
Zhu et al. [174]	(1) Relative local intensity (2) Shape irregularity (3) Orientation consistency	Ultrasound	144	(1) One important microenvironment inside the tumor is vasculature, which has been classified in this paper.

Liu et al. [175]	—	Ultrasound	—	(1) Iterated Laplacian regularization based semisupervised algorithm for robust feature selection (Iter-LR-CRFS) utilized. (2) The archived Accuracy and Sensitivity are 89.0 ± 3.6% and 91.0 ± 5.2%.
